# Inhibition of TFAM-Mediated Mitophagy by Oroxylin A Restored Sorafenib Sensitivity Under Hypoxia Conditions in HepG2 Cells

**DOI:** 10.3390/ph17121727

**Published:** 2024-12-20

**Authors:** Shufan Ji, Xuefen Xu, Yujia Li, Sumin Sun, Qiuyu Fu, Yangling Qiu, Shuqi Wang, Siwei Xia, Feixia Wang, Feng Zhang, Ji Xuan, Shizhong Zheng

**Affiliations:** 1 Jangsu Key Laboratory for Pharmacology and Safety Research of Chinese Materia Medica, School of Pharmacy, Nanjing University of Chinese Medicine, Nanjing 210023, China; 20210821@njucm.edu.cn (S.J.); 460103@njucm.edu.cn (X.X.); 18260028677@163.com (Y.L.); 18861013911@163.com (S.S.); dgdfqy521@163.com (Q.F.); ylqiu720@163.com (Y.Q.); 15765339936@163.com (S.W.); 20223116@njucm.edu.cn (S.X.); 300585@njucm.edu.cn (F.W.); zhangfeng2013@njucm.edu.cn (F.Z.); 2 Department of Pharmacology, School of Medicine, Nanjing University of Chinese Medicine, No.138, Xianlin Road, Nanjing 210023, China; 3 Department of Gastroenterology, Jinling Clinical Medical College, Nanjing University of Chinese Medicine, Nanjing 210002, China

**Keywords:** Oroxylin A, TFAM, p53, sensitivity, hypoxia

## Abstract

**Background:** Liver cancer treatment encounters considerable therapeutic challenges, especially because hypoxic microenvironments markedly reduce sensitivity to chemotherapeutic agents. TFAM (mitochondrial transcription factor A) plays a crucial role in maintaining mitochondrial function. Oroxylin A (OA), a flavonoid with potential therapeutic properties, demonstrated prospects in cancer treatment. However, the mechanism of the sensitizing effect of OA on cancer cells has not been elucidated. **Methods:** MTT assays were utilized to evaluate a hypoxia-induced resistance model. Plate colony formation assays, TEM, and JC-1 staining were used to examine the effects of siTFAM on proliferation and mitochondrial damage of HepG2 cells. Cox8-EGFP-mCherry plasmid transfection, LysoTracker and MitoTracker colocalization analysis, and WB were conducted to evaluate the influence of OA on mitophagy. The effect of OA on p53 ubiquitination levels was investigated by Co-IP and the CHX chase assay. A mouse xenograft tumor model was utilized to assess the therapeutic effect of OA on HepG2 cells in vivo. **Results:** OA significantly improved the inhibitory effect of sorafenib by inhibiting mitophagy on HepG2 cells in in vitro and in vivo models. Notably, the molecular docking and thermal shift assays indicated a clear binding of OA and TFAM. Further research revealed that OA suppressed p53 acetylation and promoted its degradation by downregulating TFAM expression, which ultimately inhibited mitophagy in hypoxia. **Conclusions:** OA has demonstrated the potential to enhance the efficacy of sorafenib treatment for liver cancer, and TFAM may be one of its targets.

## 1. Introduction

The emergence of chemotherapy resistance has become a significant obstacle to effective cancer treatment [1]. It is a critical factor contributing to relapse and mortality in cancer patients. Tumor resistance mechanisms are complex and multifaceted and are broadly classified into genetic and non-genetic categories [2,3]. The development of targeted drugs was initially regarded as a breakthrough in overcoming tumor resistance. By targeting oncogenes or critical signaling pathways, these therapies have substantially prolonged survival in some patients. However, resistance to targeted drugs, such as tyrosine kinase inhibitors (TKIs) [4,5] and immune checkpoint inhibitors (ICIs) [6], almost inevitably developed during treatment. Sorafenib, a multi-targeted TKI, has shown significant efficacy in the treatment of solid tumors such as liver cancer [7]. Despite these advances, its long-term effectiveness is severely constrained by the development of resistance [8]. The mechanisms underlying sorafenib resistance involve epigenetics [9], stemness [10] and mesenchymal features [11], transport processes [12], and the tumor microenvironment [13]. To date, studies have focused on overcoming sorafenib resistance through various approaches, including combination therapy strategies, the development of novel agents, and the identification of actionable targets within the tumor microenvironment [14]. However, overall clinical efficacy has yet to be achieved. Therefore, understanding the resistance mechanisms and finding ways to enhance the sensitivity of tumor cells to sorafenib has become an area of growing interest.

Hypoxia is a characteristic feature of solid tumors resulting from the absence or abnormality of the vascular system within the tumor microenvironment [15]. It is a major cause of mitochondrial damage or dysfunction, and the activation of mitophagy at this time is an important mechanism for cellular self-regulation. Mitophagy is recognized as a double-edged sword [16]. On one hand, mitophagy maintains cellular homeostasis by removing damaged or dysfunctional mitochondria, thus supporting normal cellular metabolism and energy production [17]. On the other hand, during chemotherapy and radiotherapy, mitophagy might assist cancer cells in eliminating damaged mitochondria caused by the treatment, which could reduce the effectiveness of the therapy and contribute to tumor progression and resistance [18,19]. In the process of mitophagy, the PINK1/Parkin, BNIP3L, and FUNDC1-mediated mitophagy pathways act as key regulators and are involved in the development and progression of various malignancies. Reports suggested that hypoxia-induced mitophagy led to sorafenib resistance through the ATAD3A-PINK1/PARKIN axis in HCC [20]. Lon-induced mitophagy contributed to hypoxic resistance by stabilizing the FUNDC1-ULK1 complex at the EMC site [21]. Thus, targeting mitophagy as an effective therapeutic approach might restore the sensitivity of cancer cells to chemotherapy drugs [22].

TFAM, a protein encoded by nuclear genes, is mainly responsible for regulating the transcription, replication, and damage repair of mitochondrial DNA [23]. Inhibiting TFAM improved the efficacy of chemotherapeutic drugs by modulating mitochondrial functions [24]. Studies have shown that the loss of TFAM led to lysosomal damage, which in turn hindered mitophagy pathways, a process crucial for cellular energy balance and metabolic stability [25]. p53, as a tumor suppressor factor, is involved in various cellular processes, including cell cycle arrest, apoptosis, senescence, and DNA damage repair [26]. Acetylation and ubiquitination are crucial post-translational modifications that regulate the stability and activity of p53 [27]. Acetylation of p53 is a key aspect of its functional activity [28], as it influences protein stability by competing with ubiquitination at the same lysine sites [29]. The inhibition of acetylation at specific lysine sites of p53 enhanced its ubiquitination, leading to accelerated degradation of p53 [30]. Further research is needed to determine whether TFAM regulation in tumor cell sensitivity is related to p53.

Oroxylin A (OA) is a flavonoid compound with potential in cancer treatment [31]. Recently, the chemosensitizing effect of OA has attracted widespread attention due to its safety and non-toxicity. OA reversed imatinib resistance in chronic myeloid leukemia (CML) by suppressing Stat3 within the bone marrow microenvironment [32]. Additionally, OA restored temozolomide sensitivity in Fibronectin-induced glioma by inhibiting the IP3R1/AKT/β-catenin pathway [33]. Our objective was to explore the sensitizing effect of OA on the hypoxia-induced resistance model. Moreover, we discussed the critical role of TFAM in the enhanced sensitivity of HepG2 cells mediated by OA.

## 2. Results

### 2.1. TFAM Contributed to Hypoxia-Induced Resistance in HepG2 Cells

**Figure 1:**
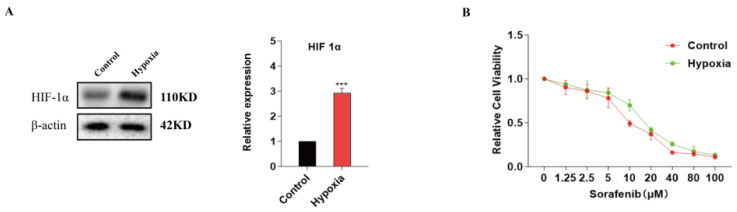

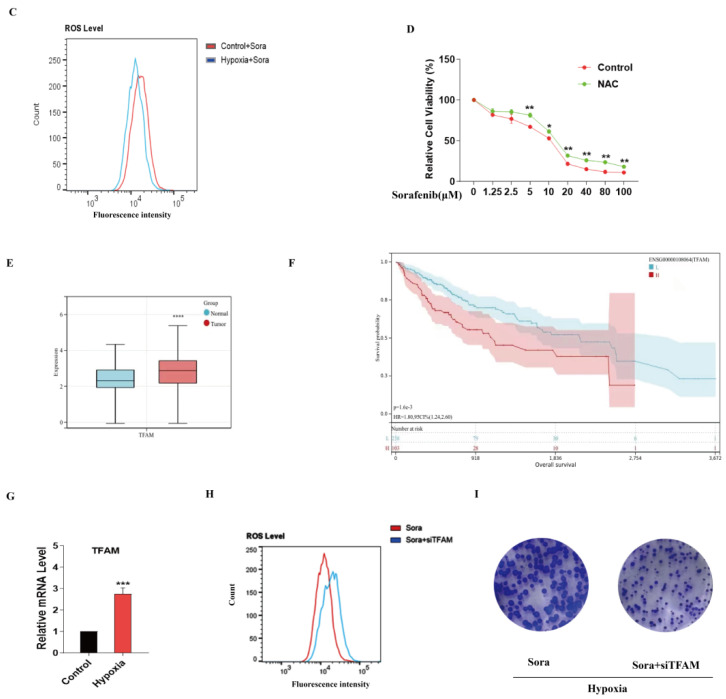
TFAM contributed to hypoxia-induced resistance in HepG2 cells. (**A**) HepG2 cells were cultured in complete medium containing 50 μM CoCl_2_ to establish a hypoxia model. HIF1α levels were assessed and analyzed by Western blot in the presence or absence of CoCl_2_ solution. (**B**) After treatment with sorafenib for 24 h, the impact of sorafenib on HepG2 cell viability was evaluated using an MTT assay under hypoxic and normoxic conditions, respectively. (**C**) DCFH-DA flow cytometry was employed to measure ROS levels in the presence of sorafenib in hypoxic and normoxic environments. ROS levels under normoxic conditions as a control. (**D**) With the addition of 2 mmol/L NAC for 1 h under normal conditions, the influence of sorafenib on HepG2 cell viability was determined via the MTT assay. (**E**) The TCGA database was analyzed to identify differences in TFAM expression between liver cancer tissues and adjacent non-tumor tissues. (**F**) Patients with high TFAM expression in liver cancer exhibited worse prognosis. (**G**) RT-PCR showed that TFAM mRNA significantly increased in HepG2 cells under hypoxic conditions. GAPDH mRNA was used to normalize the mRNA level of each gene. (**H**) TFAM was knocked down by TFAMsiRNA, then HepG2 cells were treated with 10 μM sorafenib for 24 h under hypoxic conditions. DCFH-DA flow cytometry revealed that knockdown of TFAM enhanced the ROS levels in sorafenib-treated HepG2 cells in the resistance model. (**I**) The plate cloning assay showed that TFAM siRNA significantly decreased colonies of HepG2 cells treated with sorafenib for 24 h under hypoxic conditions. Data are expressed as mean ± SEM. * *p* < 0.05, ** *p* < 0.01, and *** *p* < 0.001 versus control.

Sorafenib is known to enhance intracellular ROS levels by targeting the mitochondrial electron transport chain to promote oxidative stress. However, emerging evidence suggested that a hypoxic microenvironment could induce drug resistance in tumor cells. To explore its underlying mechanism, we established a hypoxia-induced resistance model by subjecting cells to prolonged exposure to 50 μM CoCl_2_ solution. As shown in Figure 1A, the elevated expression of HIF-1α was observed in HepG2 cells in a hypoxic microenvironment. After addition of sorafenib for 24 h, MTT assays showed that the inhibitory effect of sorafenib on HepG2 cells was significantly diminished at various concentrations compared to normal conditions (Figure 1B). Meanwhile, ROS levels in HepG2 cells were assessed following sorafenib treatment in normoxic and hypoxic microenvironments, respectively. We observed lower ROS levels under hypoxia conditions (Figure 1C), indicating that the decreased ROS could be a compensatory mechanism in response to sorafenib intervention within a hypoxic microenvironment. To verify this hypothesis, the ROS inhibitor (NAC) was applied in normoxia. The results showed that 2 mmol/L NAC reduced the sensitivity of HepG2 cells to sorafenib (Figure 1D). The above results indicated that overcoming ROS reduction might be key to reversing hypoxia-induced resistance in HepG2 cells. Subsequently, analysis of liver cancer sample data from the TCGA database revealed a significant upregulation of TFAM in patients (Figure 1E). As illustrated in Figure 1F, overall survival analysis further indicated that there was a negative correlation between TFAM and survival rate in liver cancer patients. Consistent with this, a significant increase in TFAM mRNA was observed in HepG2 cells within the hypoxia-induced resistance model (Figure 1G). To further verify the relationship between TFAM and drug sensitivity, TFAM was silenced by TFAM siRNA in HepG2 cells. Our results revealed that silencing TFAM led to a significant increase in ROS levels in sorafenib-treated HepG2 cells under hypoxia conditions (Figure 1H). Plate cloning assays also demonstrated that the combination of TFAM siRNA and sorafenib significantly reduced the colonies of HepG2 cells within a hypoxic microenvironment (Figure 1I). These findings suggested that TFAM might play a crucial role in chemotherapeutic drug resistance in tumor.

### 2.2. Silencing TFAM Enhanced the Sensitivity of HepG2 Cells to Sorafenib by Inhibiting Mitophagy

**Figure 2:**
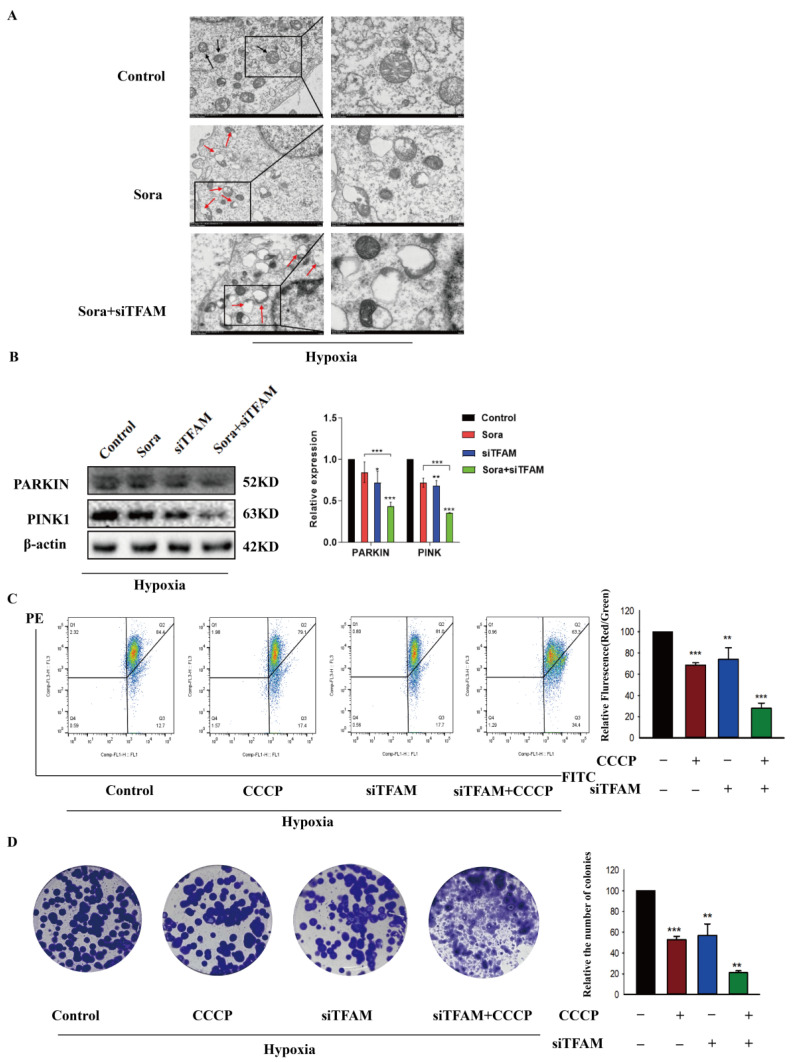
Silencing TFAM enhanced the sensitivity of HepG2 cells to sorafenib by inhibiting mitophagy. The changes in mitochondria were observed after sorafenib intervention for 24 h in HepG2 cells, with or without TFAM knockdown. (**A**) Upon TFAM knockdown, the mitochondrial morphology was detected by TEM in sorafenib-treated HepG2 cells under hypoxia conditions. The black arrows indicated normal mitochondria, and the red arrows indicated the damaged mitochondria. The left scale bar: 2.0 μm; the right scale bar: 500 nm. (**B**) Mitophagy-related proteins (parkin and pink1) were analyzed by Western blot. (**C**) Flow cytometry measured the effect of TFAM knockdown on MMP levels in hypoxia. HepG2 cells were treated with 5 μM CCCP for 24 h. The experiment was repeated three times. (**D**) Plate cloning assays showed that combination of 5 μM CCCP and TFAM knockdown under hypoxia conditions significantly promoted the inhibitory effect on cell proliferation. Data are expressed as mean ± SEM, where * *p* < 0.05, ** *p* < 0.01, and *** *p* < 0.001 denote statistical significance.

To further explore how silencing TFAM restored the sensitivity of HepG2 cells to sorafenib, intracellular mitochondria were observed. TEM showed that the combination of TFAM siRNA and sorafenib significantly increased mitochondrial damage compared to sorafenib alone in hypoxia (Figure 2A). Mitophagy was the primary protective mechanism for repairing damaged mitochondria, and it compensatorily increased in response to mitochondrial damage [34]. Interestingly, we found that silencing TFAM actually inhibited this protective mechanism in HepG2 cells under hypoxic conditions (Figure 2B). Next, flow cytometry demonstrated that TFAM siRNA reduced MMP, similar to the effect observed with CCCP, suggesting an increase in mitochondrial damage (Figure 2C). As shown in Figure 2D, administration of CCCP and TFAM siRNA individually could both decreased the number of HepG2 cell colonies. Combining CCCP with TFAM siRNA significantly reduced the number of cell colonies. This suggested that downregulation of TFAM promoted sorafenib toxicity by inhibiting mitophagy, reversing hypoxia-induced resistance in HepG2 cells.

### 2.3. OA Enhanced the Sensitivity of HepG2 Cells to Sorafenib by Inhibiting Mitophagy

**Figure 3:**
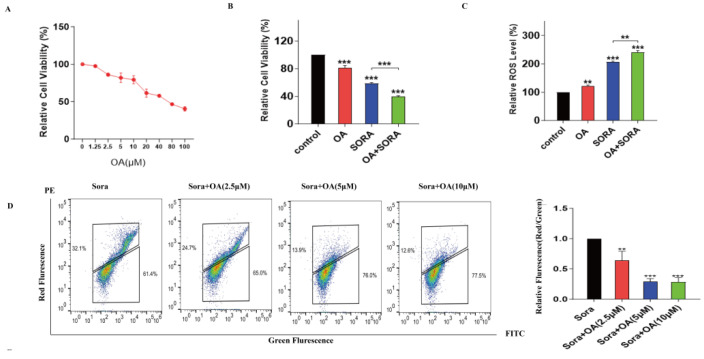

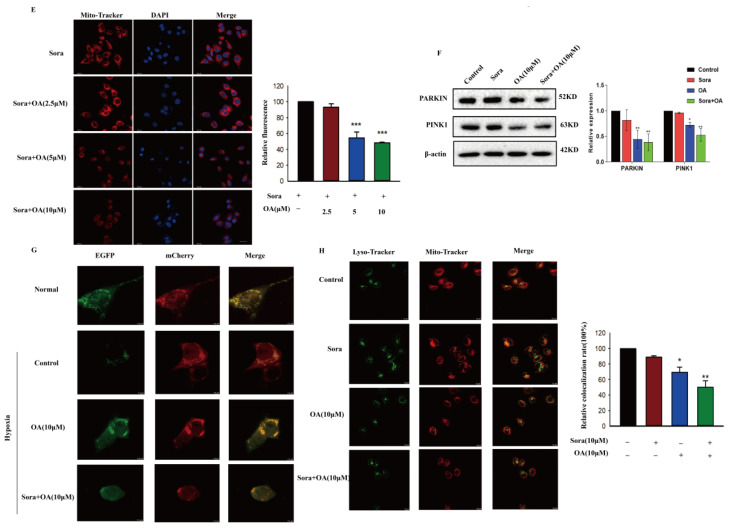
OA enhanced the sensitivity of HepG2 cells to sorafenib by inhibiting mitophagy. HepG2 cells were treated with different concentrations of OA or 10 μM sorafenib for 24 h under hypoxia conditions. (**A**) The MTT assay determined the effect of OA on the viability of HepG2 cells. (**B**) The inhibitory effects of OA (10 μM) and sorafenib (10 μM), either individually or in combination, on HepG2 cells were evaluated under hypoxia conditions. (**C**) The DCFH-DA assay determined the effect of OA (10 μM) and sorafenib (10 μM), either individually or in combination, on ROS levels. (**D**) Flow cytometry detected the effect of OA (10 μM) and sorafenib (10 μM), either individually or in combination, on MMP levels. (**E**) The mitochondrial count was evaluated using Mito-Tracker Red CMXRos after individual or combined treatments. Scale bar: 10 μm. (**F**) The levels of mitophagy-related proteins were determined by Western blot after individual or combined treatments. (**G**) HepG2 cells were transiently transfected with the Cox8-EGFP-mCherry plasmid and subsequently treated with OA, either alone or in combination with sorafenib for 24 h. The Cox8-EGFP-mCherry dual fluorescence reporter system was analyzed using confocal microscopy. Both OA treatment alone and the combined treatment with sorafenib markedly enhanced the green fluorescence intensity (EGFP). Scale bar: 2.5 μm. (**H**) Laser confocal microscopy assessed the colocalization of mitochondria and lysosomes. Scale bar: 10 μm. Data are expressed as mean ± SEM, where * *p* < 0.05, ** *p* < 0.01, and *** *p* < 0.001 denote statistical significance.

Given the previous findings, our study aimed to explore whether OA could effectively improve the therapeutic effect of sorafenib within a hypoxic microenvironment. Firstly, to assess the effect of OA on cell proliferation, HepG2 cells were treated with OA at various concentrations for 24 h. The MTT assay showed that OA exhibited a significant inhibitory effect on HepG2 cells in a concentration-dependent manner in hypoxia (Figure 3A). As shown in Figure 3A, at concentrations of 10 μM, the inhibition rate of OA was less than 20% under hypoxia-induced resistance conditions. In order to minimize the effect on the proliferation of HepG2 cells, we selected concentrations of OA of 2.5, 5, and 10 μM for the subsequent experiments. An OA concentration of 10 μM significantly enhanced the inhibition of sorafenib on HepG2 cells within the hypoxia model (Figure 3B). Meanwhile, elevated intracellular ROS was detected in the combination group (Figure 3C). The analysis of flow cytometry further indicated that OA could reduce the MMP level in HepG2 cells in the presence of sorafenib in a concentration-dependent manner (Figure 3D). Consistent with this finding, a corresponding decrease in the number of mitochondria in HepG2 cells was observed by Mitotracker staining after treating with the combination for 24 h (Figure 3E). These results indicated that OA enhanced the damaging effects of sorafenib on the mitochondria. Subsequently, the effect of combination on mitophagy levels was investigated under hypoxia-induced resistance conditions in HepG2 cells. The combination could markedly suppress the expression of autophagy-related proteins pink1 and parkin (Figure 3F). Mitophagy was subsequently assessed using dual fluorescence Cox8-EGFP-mCherry. As shown in Figure 3G, following transfection of HepG2 cells with the Cox8-EGFP-mCherry plasmid, OA significantly enhanced green fluorescence (EGFP) compared to the control group. When combined with sorafenib, 10 μM OA similarly increased green fluorescence (EGFP), while red fluorescence (mCherry) was markedly reduced. Moreover, the level of mitophagy in hypoxia was significantly higher than under normoxic conditions. The above results indicated that OA might enhance sorafenib-induced mitochondrial damage through the inhibition of mitophagy, ultimately reversing drug resistance in hypoxia. Additionally, confocal microscopy demonstrated that 10 μM OA obviously decreased the colocalization levels of mitochondria and lysosomes in HepG2 cells (Figure 3H). The combination also demonstrated this effect. The findings further validated our above conclusions.

### 2.4. OA Targeted TFAM to Inhibit Mitophagy in HepG2 Cells

**Figure 4:**
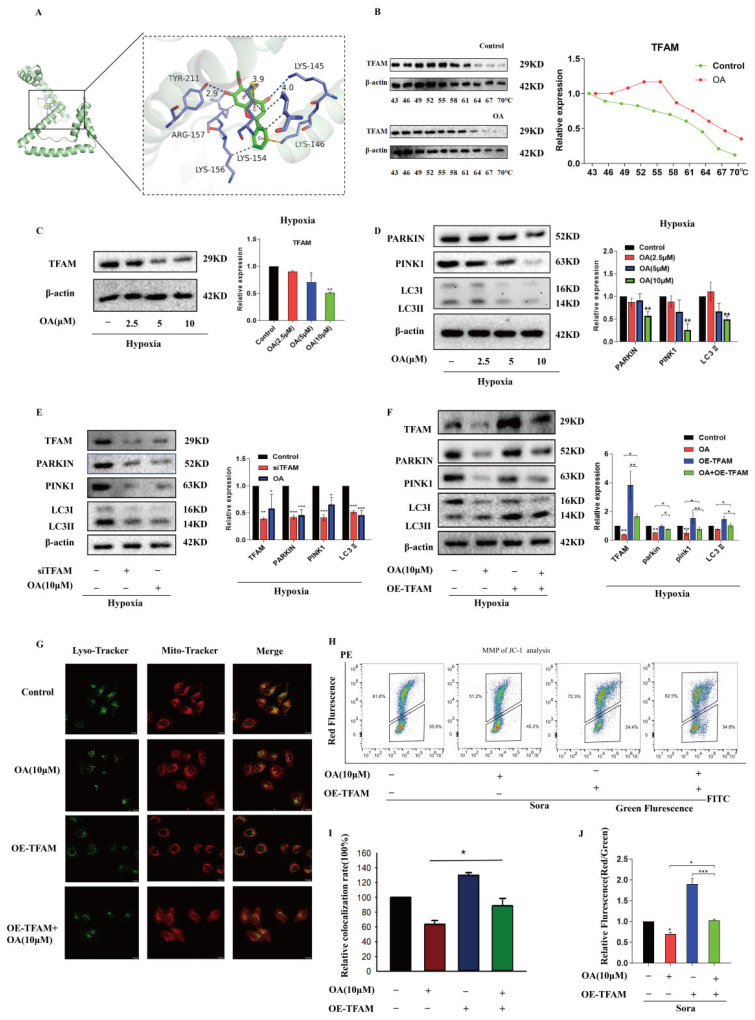
OA targeted TFAM to inhibit mitophagy in HepG2 cells. HepG2 cells were treated with OA and 10 μM sorafenib, either individually or in combination, for 24 h under hypoxic conditions. (**A**) Molecular docking results showed a binding pocket between OA and TFAM in the 3D structure. OA formed hydrogen bonds with TYR211 and LYS145 of TFAM with hydrogen bond lengths of 2.9 and 4.0, respectively. The compound formed hydrophobic interactions with ARG157 and LYS156 of TFAM and π–cation interactions with LYS154 and LYS146 of the protein. (**B**) Thermal shift assays showed that treatment with OA decreased the degradation rate of TFAM. The thermal melting curve displayed a significant rightward shift following the administration of OA. (**C**) The effects of OA on TFAM expression were determined and analyzed by Western blot in hypoxia-induced resistance. (**D**) OA reduced the expression of mitophagy-related proteins in a concentration-dependent manner, which was detected by Western blot. (**E**) Western blot revealed that either knocking down TFAM or using OA reduced mitophagy-related proteins in HepG2 cells under hypoxia conditions. (**F**) Western blot analysis indicated that overexpression of TFAM could reverse the OA-mediated inhibition of mitophagy in hypoxia-induced resistant HepG2 cells. (**G**,**I**) Laser confocal microscopy analysis revealed that TFAM overexpression could counteract the OA-mediated suppression of mitophagy under hypoxia conditions. Scale bar: 10 μm. (**H**,**J**) Flow cytometry analysis showed that overexpression of TFAM could reverse OA-induced downregulation of MMP level in HepG2 cells. Data are expressed as mean ± SEM, where * *p* < 0.05, ** *p* < 0.01, and *** *p* < 0.001 denote statistical significance.

Based on the above results, we confirmed that silencing TFAM contributed to the inhibition of mitophagy in HepG2 cells within the resistance model. Thus, we had reason to hypothesize that the sensitizing effect of OA on HepG2 cells might be related to TFAM under hypoxia-induced resistance conditions. To confirm this hypothesis, we first employed molecular docking technology. OA and TFAM could effectively form an active binding pocket with a binding energy of −6.1 kcal/mol (Figure 4A). Interaction analysis in the 3D structure revealed that OA formed hydrogen bonds with TYR211 and LYS145 of TFAM with hydrogen bond lengths of 2.9 and 4.0, respectively. Additionally, the compound formed hydrophobic interactions with ARG157 and LYS156 of TFAM and π–cation interactions with LYS154 and LYS146 of the protein (Figure 4A). Additionally, as illustrated in Figure 4B, thermal shift assays showed a significant rightward shift in the thermal melting curve upon OA administration, indicating the interaction between OA and TFAM.

Subsequently, to further verify the hypothesis, the effect of OA on TFAM was examined in HepG2 cells under hypoxia-induced resistance conditions. After administration of OA, there was a notable concentration-dependent reduction in TFAM expression in HepG2 cells (Figure 4C). Correspondingly, OA and TFAM siRNA significantly decreased mitophagy-related protein in HepG2 cells (Figure 4D,E, respectively). However, overexpression of TFAM could partially reverse OA-induced reduction in mitophagy-related protein (Figure 4F) and the colocalization levels between mitochondria and lysosomes (Figure 4G,I). Moreover, flow cytometric analysis demonstrated that overexpression of TFAM could restore the MMP level after the combination treatment (Figure 4H,J), mitigating the damaging effects of the combination therapy on mitochondria.

### 2.5. OA Suppressed Mitophagy by Downregulating TFAM to Reduce p53 Acetylation Under Hypoxia Conditions

**Figure 5:**
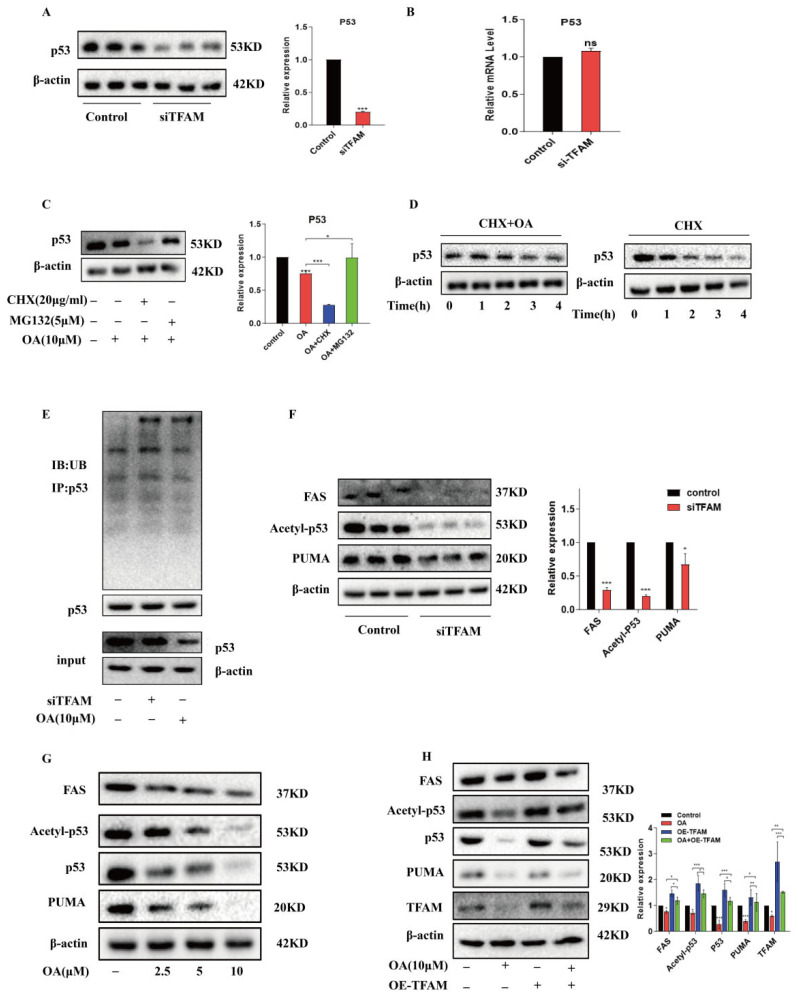
OA suppressed mitophagy by downregulating TFAM to reduce p53 acetylation under hypoxia conditions. (**A**) Western blot showed that knocking down TFAM could reduce p53 expression under hypoxia conditions. (**B**) RT-PCR analysis was used to determine the levels of p53 mRNA in HepG2 cells after TFAM knockdown. (**C**) Western blot assessed the effects of combining CHX or MG132 with OA on p53 protein. (**D**) After treatment with CHX in the presence or absence of OA, Western blot analysis evaluated the expression of p53 protein at the indicated time. (**E**) Co-IP detected the ubiquitination levels of p53 following treatment with either TFAM knockdown or 10 μM OA for 24 h. (**F**) Western blot investigated the effects of TFAM knockdown on the expression of acetylated p53 protein and its downstream target proteins. (**G**) After treatment with OA for 24 h, Western blot assessed the impact of OA on acetylated p53 and its downstream target protein. (**H**) Western blot showed that overexpressing TFAM could reverse the effects of OA on p53 acetylation and its downstream target genes. Data are expressed as mean ± SEM, where * *p* < 0.05, ** *p* < 0.01, and *** *p* < 0.001 denote statistical significance.

p53 plays a complex and crucial dual role in autophagy regulation, which is related to its specific location within the cells [35]. To further investigate the mechanism underlying the OA-mediated inhibition of mitophagy, we measured p53 protein and mRNA levels in HepG2 cells with TFAM silenced. It was worth noting that silencing TFAM significantly decreased p53 protein expression (Figure 5A), whereas, TFAM knockdown had little impact on p53 mRNA expression (Figure 5B). This suggested that the regulation of p53 by TFAM might occur at a post-transcriptional level. By employing MG-132 (a selective 26S proteasome inhibitor) and Cycloheximide (CHX, protein synthesis inhibitor), we observed a partial restoration of OA-reduced p53 expression in administration of MG-132 (Figure 5C). The CHX tracking assays further demonstrated that OA reduced the half-life of the p53 protein (Figure 5D). Additionally, with OA or siTFAM intervention, p53 ubiquitination levels were significantly elevated in HepG2 cells (Figure 5E). The above results indicated that p53 degradation might be the primary reason for OA-induced p53 reduction. p53 acetylation and ubiquitination shared the same lysine sites, and the reduction in p53 acetylation led to an increase in its ubiquitination. Given that p53 acetylation primarily regulated the transcription of downstream target genes, we separately investigated p53 acetylation levels following TFAM knockdown and treatment with OA. Upon silencing TFAM, we observed a reduction in p53 acetylation and the expression of downstream target genes (FAS and PUMA) (Figure 5F). Similar trend effects were observed with OA (Figure 5G). On the other hand, overexpressing TFAM could reverse these effects (Figure 5H). This study indicated that OA promoted TFAM-mediated p53 degradation by reducing p53 acetylation and ultimately inhibited the expression of p53 and its downstream target genes in HepG2 cells.

### 2.6. OA Enhanced the Therapeutic Effect of Sorafenib on Xenograft Tumor In Vivo

**Figure 6:**
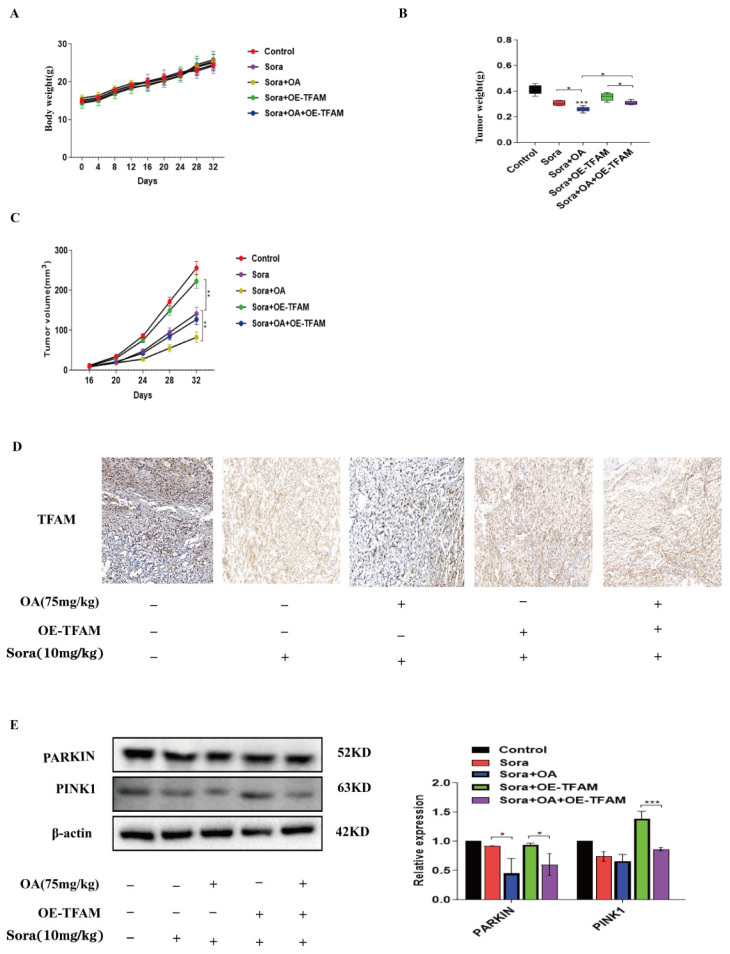
OA enhanced the therapeutic effect of sorafenib on xenograft tumor in vivo. (**A**) Changes in body weight of mice in each group. (**B**) Quantification of tumor weight in each group. (**C**) Volume changes of tumors in each group. (**D**) Immunohistochemical analysis of TFAM expression in tumor tissues of mice in each group. (**E**) Western blot analysis of the expression of mitophagy-related proteins in tumor tissues. Data are expressed as mean ± SEM, where * *p* < 0.05, ** *p* < 0.01, and *** *p* < 0.001 denote statistical significance.

We selected male nude mice (BALB/C-nu/nu) as experimental subjects and constructed a mouse xenograft tumor model by subcutaneously injecting HepG2 cells or TFAM-overexpressed HepG2 cells. As illustrated in Figure 6A, all nude mice survived during the experiment, and there were no significant changes in body weight among the different groups. The results showed that compared to using sorafenib alone, the combined treatment more effectively inhibited the growth of xenograft tumors, including significantly reducing the tumor weight and volume (Figure 6B,C). However, when TFAM was overexpressed, the inhibitory effect in the combination treatment was significantly diminished (Figure 6B,C). Immunohistochemical analysis in tumor tissues also demonstrated that the combination significantly reduced the expression of TFAM, and this reduction could be reversed when TFAM was overexpressed (Figure 6D). Additionally, as shown in Figure 6D, tumor growth was positively correlated with TFAM expression levels, consistent with the experimental results in vitro. The results of the Western blot revealed that the combination significantly reduced mitophagy-related proteins in tumor tissues, and overexpression of TFAM hindered this reduction (Figure 6E).

## 3. Discussion

Considering the urgent need to improve sorafenib sensitivity, this study investigated the potential effects of OA on HepG2 cells. Primary liver cancers include hepatocellular carcinoma (HCC), intrahepatic cholangiocarcinoma (ICCA), hepatoblastoma (HB), and angiosarcoma. The human hepatoma HepG2 cell line, also classified as hepatoblastoma (HB), represents an intermediate state between normal hepatocytes and tumor cells, exhibiting significant epigenetic alterations in the regulation of nuclear and mitochondrial genes [36,37]. Comprehensive studies at the genomic, transcriptomic, and proteomic levels have revealed that certain gene mutations in HepG2, such as TP53 and CTNNB1, coincided with those found in HCC and HB [38]. Therefore, our research might provide guidance for sensitization strategies in liver cancer. Hypoxia had a dual effect on mitochondria. On one hand, it induced changes such as redox reprogramming and increased ROS production, which lead to mitophagy [39]. On the other hand, hypoxia has also been found to promote mitochondrial biogenesis through various mechanisms, including NOS and PGC-1α [40,41]. Excessive activation of mitophagy was crucial for maintaining the integrity of these essential organelles [42]. Mitophagy, playing an essential housekeeping role, removed dysfunctional or excess mitochondria in response to stress conditions such as hypoxia and drug intervention [43]. PINK1/PARKIN-dependent mitophagy is the primary mechanism for eliminating depolarized mitochondria in cells. Therefore, further studies on mitophagy will provide new therapeutic prospects for enhancing sensitivity to sorafenib. As expected, the mitophagy markers PINK1, PARKIN, and LC3 in HepG2 cells showed significant changes after treatment with OA. Concurrently, OA combined with sorafenib also promoted mitochondrial damage and the accumulation of intracellular ROS.

In this study, we observed that TFAM played a crucial role in maintaining mitochondrial function. Firstly, by analyzing the TCGA database, the results indicated that the expression of TFAM was directly associated with the advancement of liver cancer. Our findings confirmed that knocking down TFAM promoted sorafenib-induced mitochondrial damage by increasing ROS levels and inhibited the growth of HepG2 cells. Notably, although mitophagy was responsible for repairing mitochondrial damage, our research indicated that reducing TFAM actually suppressed this protective process in HepG2 cells, implying that the decreased TFAM may worsen mitochondrial damage by inhibiting mitophagy.

Subsequently, we focused on exploring the potential of OA to reverse hypoxia-induced resistance in HepG2 and elucidating the underlying mechanisms. We found that, at safe doses, OA significantly enhanced the inhibitory effect of sorafenib on cell viability. Further experiments indicated that the restoration of sensitivity by OA was associated with mitophagy in HepG2 cells. After treatment with the combination, Mitotracker staining revealed a significant reduction in mitochondrial number. Additionally, mitophagy-related proteins PINK1 and Parkin were also markedly decreased. Based on these findings, we hypothesized that the inhibition of mitophagy by OA might be linked to the regulation of TFAM. Molecular docking and thermal shift assays also demonstrated that OA could bind to TFAM with lower binding energy in vitro. To further confirm our hypothesis, we continued to conduct molecular biology experiments both in vivo and in vitro. Interestingly, OA significantly reduced TFAM expression in HepG2 cells. Treatment with OA alone decreased mitophagy in a concentration-dependent manner, and overexpression of TFAM could reverse this reduction. TFAM plays a key role in maintaining the stability and transcriptional activity of mitochondrial DNA. The targeted binding of OA to TFAM might decrease TFAM transcription levels and downregulate TFAM expression. OA inhibited mitophagy by reducing TFAM, which supported the idea that TFAM is essential for in mitochondrial quality control when the combination of sorafenib and OA might potentially exacerbate mitochondrial dysfunction and oxidative stress, which could create a synergistic effect, enhancing tumor cell death. While our study presented strong evidence linking TFAM with the sensitization effect of OA, alternative pathways that might contribute to the observed effects were also considered. As a targeted therapy for liver cancer, sorafenib exerted its anti-tumor effects by triggering ferroptosis and/or apoptosis through distinct mechanisms. While we observed an increase in cell death when Oroxylin A was combined with sorafenib, the precise mode of cell death remained to be elucidated.

In the nucleus, p53 could promote autophagy through transcription-dependent or -independent mechanisms. When located in the cytoplasm, p53 shifted its role to inhibiting autophagy [44]. Recent studies had revealed that a decrease in TFAM expression not only affected the stability and acetylation of p53 but was also accompanied by a reduction in PISD and the inhibition of autophagy [45]. Our research found that OA suppressed mitophagy by downregulating the TFAM/p53 pathway under hypoxia-induced resistance conditions. Further studies revealed that OA reduced p53 acetylation and increased its ubiquitination by inhibiting TFAM, which ultimately led to a decrease in p53 protein levels. This was consistent with previous reports that knocking down TFAM decreased p53 expression and affected mitophagy level [46]. Our findings suggested that following OA’s entry into the cell, it first bound to TFAM and then acted on cytoplasmic p53, leading to changes in p53 post-translational modifications and subsequently inhibiting mitophagy. These differences in effects of p53 might be related to the characteristics of the cells themselves and their microenvironment. In summary, these data indicated that OA had potential anticancer activity by targeting TFAM and eliminating drug-resistant cells in hypoxic microenvironments in liver cancer. A limitation of this study was the exclusive use of the HepG2 cell line, which might limit the generalizability of the findings. Future studies should focus on elucidating the precise molecular interaction between OA and TFAM in other liver cancer models.

## 4. Materials and Methods

### 4.1. Cell Culture and Drug Treatment

HepG2 cells (Procell Life Science&Technology Co., Ltd., Wuhan, China) were cultured in DMEM (Servicebio, Wuhan, China) supplemented with 10% fetal bovine serum at 37 °C and 5% CO_2_. To establish a hypoxia-resistant model, HepG2 cells were continuously exposed to complete medium containing 50 μM CoCl_2_ solution. OA (provided by Jiangsu Key Laboratory of Carcinogenesis and Intervention, China Pharmaceutical University, 99%) and sorafenib were dissolved in DMSO (Beyotime, Shanghai, China) at a concentration of 10 mM and stored at −20 °C. Cells were grown to 70% confluence and then exposed to the drug at the specified concentration. NAC (N-acetylcysteine, Cat. #S0077, Beyotime, Shanghai, China), CCCP (Cat.#C2006-4, Beyotime, Shanghai, China), and sorafenib (Cat.#MB1666-1, MeilunBio, Dalian, China) were used in this study.

### 4.2. Real-Time PCR Analysis

Real-time PCR analysis was conducted with reference to the instructions provided by Yeasen Biotech Co., Ltd. (Shanghai, China). The primer (Tsingke Biotechnology Co., Ltd., Nanjing, China) is as follows in Table 1:

### 4.3. Western Blot Analysis

The acquisition of protein lysates and their transfer onto PVDF membranes (Beyotime, Shanghai, China) was conducted with reference to the previous methods. Blot visualization was achieved through enhanced chemiluminescence (GE Healthcare Life Sciences, Pittsburgh, PA, USA). Densitometry analysis was performed using ImageJ software (ImageJ 1.53). The details of the antibodies are as follows in Table 2:

### 4.4. MTT Assay

The HepG2 cell suspension was adjusted to approximately 5000 cells per well after accurate counting. Prepared drugs were added to a 96-well plate after cell adherence and incubated for 24 h. Subsequently, MTT (BioFroxx Cat.#1334GR005, Einhausen, Germany) solution (5 mg/mL in PBS) was mixed with DMEM in a 1:10 ratio, added to each well, and incubated for 4 h. After removing the supernatant, 150 μL of DMSO was added, and then cell viability was assessed by measuring absorbance at 490 nm.

### 4.5. Transmission Electron Microscopy (TEM)

TEM was employed to examine mitochondrial morphology and autophagosome quantity. Briefly, the culture medium was discarded, and electron microscopy fixative solution was directly applied. Cells were gently scraped using a cell scraper and transferred to a centrifuge tube. The following steps were carried out as previously described. Images were captured by Servicebio (Wuhan, China).

### 4.6. Cellular Thermal Shift Assay (CETSA)

To evaluate the effect of OA on the stability of TFAM protein, cells were incubated for 2 h with or without OA, then divided into ten parts and separately heated for 3 min at temperatures ranging from 43 to 70 °C. After heating, the samples were quickly frozen to −80 °C and stored for 12 h, then thawed at room temperature for 5 min, followed by repeating the freeze–thaw process. Soluble proteins were separated by high-speed centrifugation, and the TFAM levels were detected using Western blot to determine the stability of OA and the protein at different temperatures.

### 4.7. Immunohistochemistry

Immunostaining was performed as previously described. Tissues (obtained from the xenograft tumor) were dewaxed and rinsed, and sections were incubated in hydrogen peroxide block for 10–15 min to diminish non-specific background staining. Finally, sections were exposed to DAB for 10 min. Staining results were observed under a microscope.

### 4.8. Colocalization of Mitochondria and Lysosomes

Log-phase HepG2 cells were seeded into confocal dishes at a density of 1 × 10^5^ cells/well. After drug treatment for 24 h, the culture medium was removed. The cells were then incubated with Lyso-Tracker Green fluorescent probes (Beyotime, Shanghai, China) and Mito-Tracker Red CMXRos (Beyotime, Shanghai, China) following the manufacturer’s protocol. Finally, mitochondrial and lysosomal colocalization was visualized using confocal microscopy.

### 4.9. Co-Immunoprecipitation (Co-IP)

In this method, we used rProtein A/G Plus MagPoly Beads (ABclonal, Wuhan, China). We lysed cells in a protease inhibitor buffer for 10 min at 4 °C then centrifuged them at 12,000 rpm for 15 min. After quantifying the protein concentration with NanoDrop, we prepared equal volumes of lysate for Co-IP. We incubated the lysate with a p53 antibody at 4 °C for 24 h, followed by incubation with pre-treated beads for 24 h. Following three washes, we eluted the immune complexes and analyzed them by SDS-PAGE and Western blot.

### 4.10. Cox8-EGFP-mCherry to Monitor Mitophagy

Log-phase HepG2 cells were seeded into confocal dishes at a density of 1 × 10^5^ cells/well. When the cells reached approximately 60% confluence, they were transiently transfected with the Cox8-EGFP-mCherry plasmid. The following day, cells were treated with the specified drug (OA, sorafenib) for 24 h then observed using confocal microscopy. Under normal conditions, HepG2 cells expressing Cox8-EGFP-mCherry exhibit yellow-colored mitochondria. Upon induction of mitophagy, damaged mitochondria are recognized and sequestered by autophagosomes, which subsequently transport them to lysosomes for fusion, forming autolysosomes. Within the autolysosomes, the sequestered mitochondria display only red fluorescence (mCherry) due to the quenching of the green fluorescence (EGFP).

### 4.11. TCGA Database Analysis

Using the R package maxstat (version 0.7-25), we established an optimal TFAM cutoff at 3.0163 based on sample grouping ratios between 25% and 75%. Patients were categorized into high or low expression groups accordingly. Prognostic differences between these groups were analyzed using the survfit function from R’s survival package, with significance evaluated by the logrank test. This analysis revealed significant prognostic disparities between the groups (*p* = 0.02).

### 4.12. Plate Colony Formation Assay

Cells were seeded at 1000 cells/well in a 6-well plate for 24 h, followed by treatment with drugs. Following incubation for 14 days, cells underwent two PBS washes and were fixed with 4% formaldehyde (Beyotime, Shanghai, China) for 15 min. Staining was achieved using crystal violet for 15 min, with subsequent rinsing in deionized water to remove excess stain. Photographs of the stained colonies were captured for analysis.

### 4.13. The Molecular Docking

The molecular docking of hTFAM (PDB id: 3tq6) and OA (CAS: 480-11-5) was performed by using Autodock vina 1.1.2. The highest-scoring conformations were selected and visualized using Discovery Studio 4.5.

### 4.14. Animal Experiments

Male nude mice (BALB/C-nu/nu) were acquired and allowed to acclimate for one week before receiving a subcutaneous injection of either 2 × 10^7^ HepG2 cells or TFAM-overexpressing HepG2 cells, establishing a xenograft model. The mice were organized into five groups (five per group): the model group, sorafenib group, sorafenib and OA group, sorafenib and TFAM overexpression group, and sorafenib with OA and TFAM overexpression group. Upon reaching a tumor size of 50 mm^3^, 75 mg/kg OA and 10 mg/kg sorafenib were administered intraperitoneally every other day for approximately 3 weeks, individually or in combination. At the end of the experiment, all mice were anesthetized, and tumor tissues were obtained. Tumor specimens were preserved in 10% formalin for histopathological analysis. The ethics approval number for animal experiments was 202210A066.

### 4.15. Measurement of Intracellular Reactive Oxygen Species (ROS) Levels

Intracellular ROS levels were assessed utilizing DCFH-DA according to the manufacturer’s instructions (Beyotime, Shanghai, China).

### 4.16. Mitochondrial Membrane Potential (MMP) Measurement

MMP were evaluated with the JC-1 MMP Assay Kit (Beyotime, Shanghai, China).

### 4.17. Statistical Analysis

Data are presented as mean ± standard error of mean (SEM). Statistical analyses and graphical representations were conducted using GraphPad Prism 10 software. For data following a normal distribution, comparisons between two groups were performed using the *t*-test, while differences among multiple groups were assessed using one-way analysis of variance (ANOVA). All data were obtained from three independent experiments. Statistical significance was established at * *p* < 0.05.

## 5. Conclusions

Our findings demonstrated that OA significantly reduced p53 acetylation and enhanced its ubiquitination by targeting TFAM, subsequently inhibited mitophagy, and ultimately restored the sensitivity of HepG2 cells to sorafenib in a hypoxia-induced resistance model in vivo and in vitro. Our research suggested that targeting TFAM could be an effective approach to enhance the sensitivity of cancer patients to sorafenib, and OA might be a promising anti-cancer agent, especially in a hypoxic microenvironment. However, considering the limitations of this study, further research utilizing other liver cancer models will be necessary to validate these findings.

## Figures and Tables

**Table 1:** The primer of genes.

Gene (Human)	Forward Sequence	Reverse Sequence
*p53*	GATCAGCAGAGCATTGTTCACATTG	GGGTCGTCGCCTCCAGTTG
*TFAM*	CCGAGGTGGTTTTCATCTGT	TATATACCTGCCACTCCGCC
*GADPH*	ATTCCACCCATGGCAAATTCC	GACTCCACGACGTACTCAGC

**Table 2:** Source of the antibody.

Antibody	Catalog Number	Company
Anti-TFAM	AF0531	Affinity Biosciences (Liyang, China)
Anti-p53	2524T	CST (Danvers, MA, USA)
Anti-HIF 1α	AF02369	AiFang biological (Changsha, China)
Anti-Acetyl p53	Ab179484	Abcam (Cambridge, UK)
Anti-PINK1	Ab300623	Abcam (Cambridge, UK)
Anti-PARKIN	14060-1-AP	Proteintech (Wuhan, China)
Anti-LC3B	AF11004	AiFang biological (Changsha, China)
Anti-FAS	AF301026	AiFang biological (Changsha, China)
Anti-PUMA	AF300458	AiFang biological (Changsha, China)
Anti-β-actin	20536-1-AP	Proteintech (Wuhan, China)

## Data Availability

The original contributions presented in the study are included in the article, further inquiries can be directed to the corresponding authors.

## Abbreviations

HCC, hepatocellular carcinoma; TFAM, mitochondrial transcription factor A; CML, chronic myeloid leukemia; OA, Oroxylin A; sora, sorafenib; MMP, mitochondrial membrane potential; CHX, Cycloheximide; Co-IP, co-immunoprecipitation; TEM, transmission electron microscopy; NAC, N-acetylcysteine; Cellular Thermal Shift Assay (CETSA); ROS, reactive oxygen species; TKIs, tyrosine kinase inhibitors; ICIs, immune checkpoint inhibitors.
